# Heat Shock Protein 22 Attenuates Doxorubicin-Induced Cardiotoxicity *via* Regulating Inflammation and Apoptosis

**DOI:** 10.3389/fphar.2020.00257

**Published:** 2020-03-25

**Authors:** Yin Lan, Yi Wang, Kun Huang, Qiutang Zeng

**Affiliations:** ^1^Department of Cardiology, Union Hospital, Tongji Medical College, Huazhong University of Science and Technology, Wuhan, China; ^2^Department of Ultrasound, Wuhan Asia Heart Hospital, Wuhan, China

**Keywords:** doxorubicin, Hsp22, inflammation, apoptosis, TLR/NLRP3

## Abstract

**Background:**

The antitumor effect of doxorubicin (DOX) is limited by its acute and chronic toxicity to the heart, which causes heart injury. Heat shock protein 22 (Hsp22) is a protein proved to exert anti-apoptosis and anti-inflammatory effects in other diseases and physical conditions. In this study, we aim to explore whether Hsp22 could exert a protective role during cardiac injury in response to DOX.

**Methods:**

The overexpression of Hsp22 was mediated *via* adenovirus vector to clarify the role of Hsp22 in the cardiac injury caused by DOX. DOX-induced acute heart injury mouse model was established by single intraperitoneal injection of DOX (15 mg/kg). Subsequently, cardiac staining and molecular biological analysis were performed to analyze the morphological and biochemical effects of Hsp22 on cardiac injury. H9c2 cells were used for validation *in vitro*.

**Results:**

An increase in the expression level of Hsp22 was observed in DOX-treated heart tissue. Furthermore, cardiac-specific overexpression of Hsp22 showed reduced cardiac dysfunction, decrease in inflammatory response, and reduction in cell apoptosis in injury heart and cardiomyocytes induced by DOX *in vivo* and *in vitro*. Moreover, the suppression of Toll-like receptor (TLR)4/NOD-, LRR-, and pyrin domain-containing protein 3 (NLRP3) was associated with the protective effect of Hsp22. Finally, the protective effect of Hsp22 cardiac function was almost abolished by overexpression of NLRP3 in DOX-treated mice.

**Conclusion:**

In summary, Hsp22 overexpression in the heart could suppress cardiac injury in response to DOX treatment through blocking TLR4/NLRP3 activation. Hsp22 may become a new therapeutic method for treating cardiac injury induced by DOX in cancer patients.

## Introduction

Doxorubicin (DOX) is a highly effective chemotherapy drug used to treat tumors, including solid and hematopoietic tumors. However, many patients have to discontinue DOX administration due to irreversible cardiotoxicity (Octavia et al., 2012). Doxorubicin-related cardiotoxicity is a common adverse drug reaction, which includes severe arrhythmia, myocardial infarction, and left ventricular dysfunction. The incidence of adverse drug reaction is approximately 30%–40% of the patients who received DOX treatment (Ferreira et al., 2008). The acute cardiotoxicity presents transient symptoms such as arrhythmias; the chronic form can evolve into heart failure. The probability of developing congestive heart failure after doxorubicin treatment is estimated to be 1%–2%, which is significantly associated with high mortality in cancer chemotherapy patients (Yeh and Bickford, 2009; Plummer et al., 2019). So far, there is no specific treatment for heart injury induced by DOX. Therefore, it is urgent to find new ways to treat DOX-induced heart injury.

Hsp22 is a member of the small heat shock protein (SHSP) family, which is widely expressed under various pathological stimuli. Hsp22 presents high expression in a variety of normal tissues, especially highly detected in heart and skeletal muscles (Kappe et al., 2001). Hsp22 seems to play a critical role in the maintenance of cardiac cell survival, anti-ischemia injury (Lizano et al., 2013), anti-cardiac remodeling, and inflammatory reaction (Hedhli et al., 2008). Hsp22 may be a new potential strategy of cardiovascular diseases. However, whether Hsp22 could protect the heart from DOX injury remains unknown. Recent studies have found that Hsp22 can exert anti-apoptotic activity under various pathological conditions. Hyo et al. found transduced Tat-Hsp22 significantly attenuates oxidative stress-induced apoptosis by regulation of apoptosis-related protein expression levels in hippocampal neuronal cells (Kang et al., 2008). In addition, the study of Chen et al. reported that Hsp22 plays a cardioprotective role in ischemic myocardium, triggering an anti-apoptotic response in advance by activating the survival pathway (Jo et al., 2017). These studies suggest that Hsp22 may play a protective role in DOX-induced cardiac injury.

Toll-like receptor 4 (TLR4) is an important pattern recognition receptor, which is expressed on cell membrane and plays an important role in infectious and non-infectious diseases. Studies have found that TLR4 deficiency can alleviate DOX-induced cardiac function injury and myocardial injury (Chen et al., 2011). Therefore, exploring the negative regulatory factors of TLR4 may find specific drugs to protect against DOX-induced cardiac injury. In recent years, Hsp22 has been considered as a new TLR4 ligand and may be involved in the pathogenesis of rheumatoid arthritis (Riad et al., 2008). Activation of TLR4 triggers activation of the downstream NLRP3 inflammasome signal, which is involved in the inflammatory response by processing the maturation of interleukin (IL)-1β and IL-18 (Kang et al., 2016). Thus, we want to investigate whether Hsp22 could play a cardioprotective role against DOX toxicity *via* regulation of the TLR4/NLRP3 pathway.

## Methods

### Animals and Animal Model

All of the animal experimental procedures conformed to the National Institutes of Health (NIH) Guideline and were approved by the Ethics Committee of Union Hospital, Huazhong University of Science and Technology. The male C57BL/6 mice (aged 6–8 weeks and weighed 23–25 g) were from the Institute of Laboratory Animal Science, Chinese Academy of Medical Sciences (Beijing, China).

A random number table method was used for grouping. Mice were injected with adeno-associated virus (AAV)-Hsp22 or AAV-green fluorescent protein (GFP) (5 × 10^10^ viral genome particles/mouse) through the tail vein. Mice were then subjected to DOX (Sigma-Aldrich, St. Louis, MO, USA) injection (single intraperitoneal injection, 15 mg/kg per mouse) at 4 weeks after the AAV injection to establish the acute cardiac injury model.

### Echocardiography and Hemodynamic Analysis

A MyLab 30CV (Esaote) machine was used to perform echocardiography as a previous study described (Du et al., 2015). After being anesthetized with 1.5% isoﬂurane, mice were detected using a 10-MHz linear-array ultrasound transducer. A microtip catheter transducer (SPR-839, Millar Instruments, Houston, TX, USA) was used to measure hemodynamic data as previously described (Du et al., 2015). A Millar Pressure–Volume System (MPVS-400, Millar Instruments, USA) was used to analyze the results.

### Western Blotting and Quantitative Real-Time PCR

After treatment, protein (50 μg) from heart tissue and H9c2 cells harvested from RIPA lysis buffer were separated in a 10% sodium dodecyl sulfate–polyacrylamide gel electrophoresis (SDS-PAGE). A polyvinylidene fluoride (PVDF) membrane (Millipore) was used to transfer protein. After blocking with 10% non-fat milk, membranes were incubated with primary antibodies enclosing Hsp22 (3059), t-p65 (4764), p-p65^ser536^ (3033), cleaved caspase-3 (9661), Bcl-2 (4223), Bax (5023), cytochrome C (4280), NLRP3 (13158), cleaved-caspase-3 (9579), and glyceraldehyde-3-phosphate dehydrogenase (GAPDH) (2118); all were purchased from Cell Signaling Technology (Boston, MA, USA). Antibodies against TLR4 (sc30002) were purchased from Santa Cruz (Dallas, TX, USA). Membranes were incubated with secondary antibodies then with enhanced chemiluminescence (ECL) reagents (170–5061, Bio-Rad). GAPDH protein was used as a reference.

TRIzol Reagent (Invitrogen) was used to isolate total RNA. cDNA was synthesized using a commercial kit (Roche). SYBR Green (Roche) was used to amplify. GAPDH RNA level was used as a reference.

### Immunohistochemistry Staining

Tumor necrosis factor (TNF)-α and CD68 staining was performed as previously described (Du et al., 2015). Anti-TNF-α, CD45, or anti-CD68 (Abcam) were used to stain. EnVision^TM+^/horseradish peroxidase (HRP) reagent and 3,3ʹ-diaminobenzidine (DAB) were used to visualize. A light microscope (Nikon, Tokyo, Japan) was used to calculate.

### Cell Culture

H9c2 cells were cultured in Dulbecco’s modified Eagle’s medium (DMEM) with 10% fetal bovine serum (FBS). Before treatment, cells were starved for 6–8 h. The *in vitro* cardiac injury model was established by treating with DOX (1 μmol/L). Cell counting kit-8 (CCK-8) was used to detect cell viability.

### Terminal Deoxynucleotidyl Transferase dUTP Nick End Labeling Staining

Terminal deoxynucleotidyl transferase dUTP nick end labeling (TUNEL) staining was performed as previously described (Gao et al., 2015). TUNEL commercial reagent was used to detect apoptosis cells (Millipore, USA). Cell nucleus was stained with 4′,6-diamidino-2-phenylindole (DAPI). An OLYMPUS DX51 fluorescence microscope was used to get images (Tokyo, Japan). Image-Pro Plus 6.0 software was used to calculate.

### Statistical Analysis

SPSS 23.0 was used for analysis. Data were expressed as mean ± standard error of mean (SEM). Unpaired Student’s t-test was used to compare differences between two groups. Multiple comparisons among ≥3 groups were performed using one-way ANOVA. One-way ANOVA followed by the *post hoc* least significant difference test was used to analyze differences among multiple groups when ANOVA found a significant value of F and no variance in homogeneity; otherwise, Tamhane’s T2 *post hoc* test was used. *P* < 0.05 was considered statistically significant.

## Results

### Hsp22 Improved Cardiac Dysfunction Induced by DOX

**Figure 1:**
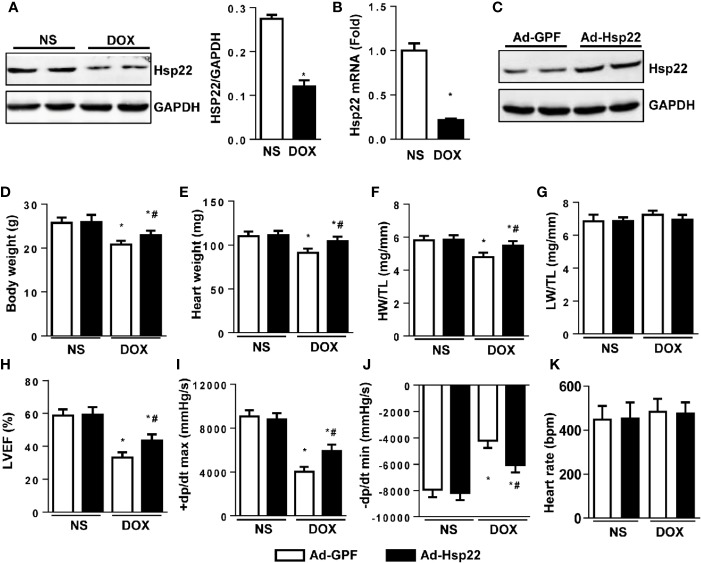
Heat shock protein 22 (Hsp22) improved cardiac function in mice with doxorubicin (DOX)-induced toxicity *in vivo*. **(A)** Representative Western blot bands and quantitative results of protein levels of Hsp22 in DOX-induced cardiac injury in C57BL/6 mice treated with DOX. **(B)** The mRNA level of Hsp22 in the C57BL/6 mice treated with DOX. **(C)** Representative Western blot bands of protein level of Hsp22 in mice injected with adeno-associated virus (AAV)-Hsp22. **(D)** Body weight of the four groups (n = 9–12). **(E)** Heart weight of the four groups (n = 9–12). **(F)** Statistical results of the heart weight (HW)/tibia length (TL) (n = 9–12). **(G)** Statistical results of the lung weight (LW)/TL (n = 9–12). **(H)** Ejection fraction (EF) of mice in the indicated groups (n = 9–12). **(I, J)** Hemodynamic analysis of mice with or without Hsp22 treatment (n = 7–11). **(K)** Heart rate of mice with or without Hsp22 treatment. *P < 0.05 versus NS + Ad-GPF, #*P* < 0.05 versus DOX + Ad-GPF. For panels **(A**, **B)**, *P* < 0.05 versus NS.

Hsp22 expression pattern was evaluated in acute cardiac injury induced by DOX. As shown in **Figures 1A, B**, Hsp22 protein and mRNA levels were lower in DOX-treated mice compared with control mice. Thus, we established Hsp22-overexpressing mice by delivering AAV, and the expression efficiency of Hsp22 is shown in **Figure 1C**. C57BL/6 mice were injected with AAV-Hsp22 or AAV-GFP through the tail vein and then subjected to DOX injection 4 weeks later.

The pathophysiological characteristics of the mice were detected. The body weight, heart weight (HW), and the ratio of HW and tibia length (TL) was higher in Hsp22 overexpression group than those in the control group after DOX injection (**Figures 1D–F**). However, the ratio of lung weight (LW) and TL showed no difference between the four groups (**Figure 1G**). Functionally, echocardiographic measurements showed a decreased ejection fraction (EF) in DOX-treated mice when compared with control mice. Conversely, left ventricular ejection fraction (LVEF) in Hsp22-overexpressing mice was higher than in AAV-GFP-injected mice (**Figure 1H**). A significant reduction in maximal rate of the increase of left ventricular pressure (+dP/dt) and the maximal rate of the decrease of left ventricular pressure (-dP/dt) in DOX-treated mice. While +dP/dt and -dP/dt were higher in Hsp22 overexpression group than AAV-GFP-injected group after DOX treatment (**Figures 1I, J**). There are reports that heart rate is affected by DOX (Robison et al., 1985); however, no significant difference in heart rate was observed among the four groups.

### Hsp22 Inhibited Inflammatory Responses in Acute Cardiac Injury Mice Model

**Figure 2:**
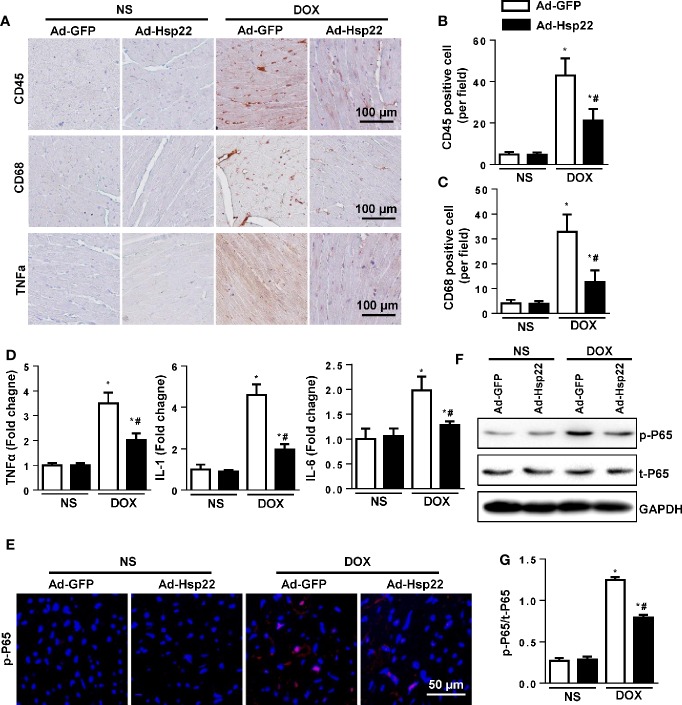
Heat shock protein 22 (Hsp22) suppressed inflammation responses in doxorubicin (DOX)-treated mice. **(A–C)** Immunohistochemistry analysis of CD45, CD68, and tumor necrosis factor (TNF)-α in DOX-treated hearts. Representative images and quantification (n = 6, 10+ fields per heart) are shown. **(D)** PCR analysis of inflammation markers [TNF-α, interleukin (IL)-1, IL-6] in DOX-treated hearts (n = 6). **(E)** Representative images of p-P65 immunofluorescence in mice with or without Ad-Hsp22 transfection (n = 4). **(F, G)** Western blot and quantitative results (n = 6). *P < 0.05 versus NS + Ad-GPF, ^#^*P* < 0.05 versus DOX + Ad-GFP.

Inflammation plays an important role in DOX-induced cardiac injury. So first, we examined the effect of Hsp22 on DOX-induced inflammatory response. As shown in **Figures 2A–C**, Hsp22 overexpression markedly decreased the number of CD45-labeled leukocyte infiltration, CD68-labeled macrophage, and the protein level of TNF-α in the DOX-treated group. Consistently, the pro-inflammatory cytokines, including TNF-α, IL-1, and IL-6, were increased in the DOX-treated group. And the inflammatory response induced by DOX was suppressed in Hsp22 overexpression group compared with mice injected with AAV-GFP (**Figure 2D**). Nuclear factor (NF)-κB is an essential molecule in inflammation during cardiac injury; thus, we evaluated NF-κB activation level (Hayden and Ghosh, 2008). An increase in NF-κB nucleus translocation was observed in the DOX-treated group, while it decreased in the Hsp22 overexpression group after DOX treatment (**Figure 2E**). Consistently, the protein expression of activated NF-κB was decreased in the AAV-Hsp22 group after DOX treatment (**Figures 2F, G**).

### Hsp22 Inhibited Cell Apoptosis in Acute Cardiac Injury Mice Model

**Figure 3:**
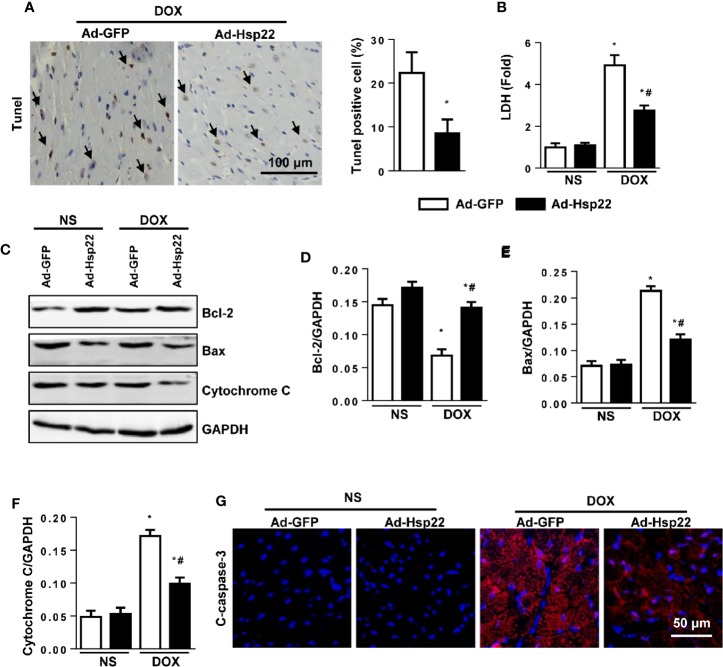
Overexpression of heat shock protein 22 (Hsp22) exhibited anti-apoptosis effect in doxorubicin (DOX)-induced cell death. **(A)** Representative images of terminal deoxynucleotidyl transferase dUTP nick end labeling (TUNEL) and the quantitative results in DOX-treated hearts (n = 4). **(B)** The level of lactate dehydrogenase (LDH) in the indicated groups (n = 6). **(C–F)** Western blot and quantitative results in the indicated groups (n = 6). **(G)** Cleaved caspase-3 (c-caspase-3) immunofluorescence (n = 4). *P < 0.05 versus NS + Ad-GFP, ^#^*P* < 0.05 versus DOX + Ad-GFP.

Cardiomyocyte apoptosis is a central mechanism underlying cardiac injury induced by DOX. Thus, we evaluated cell apoptosis. As shown in **Figure 3A**, DOX caused increased cell apoptosis in the heart; Hsp22 overexpression reduces these increased cell apoptosis. Simultaneously, we investigated the effect of DOX on the cardiomyocyte viability by measuring the release of lactate dehydrogenase (LDH). The activity of LDH was increased significantly after DOX stimulation, while Hsp22 overexpression could reduce the release of LDH (**Figure 3B**). Apoptosis-associated protein Bax and Bcl-2 were also detected, as a result, Hsp22 increased the protein level of Bcl-2 but reduced the level of Bax thus decreased the release of cytochrome C (**Figures 3C–F**). Moreover, we used cleaved caspase-3 (c-caspase-3) stain to solidify our evidence and found that Hsp22 attenuated the level of c-caspase-3 in mice heart treated with DOX (**Figure 3G**).

### Hsp22 Regulated the Expression of NLRP3 and TLR4 Both In Vitro and In Vivo

**Figure 4:**
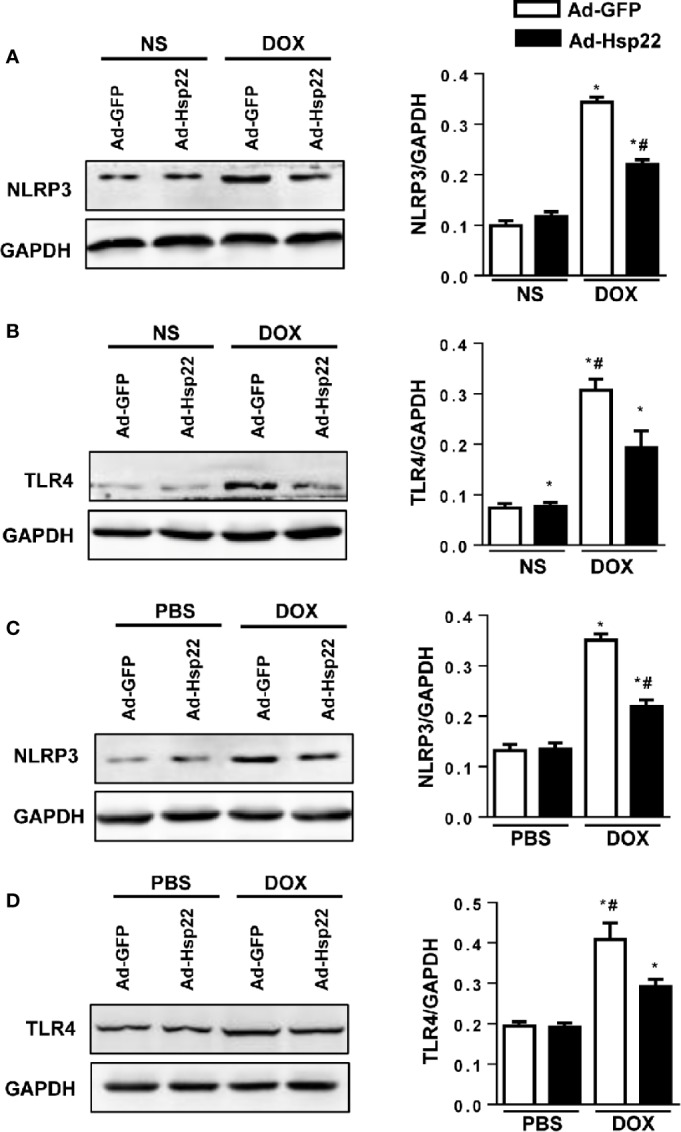
Heat shock protein 22 (Hsp22) regulated the expression of NOD-, LRR-, and Pyrin Domain-Containing Protein 3 (NLRP3) and Toll-Like Receptor (TLR)4 both *in vitro* and *in vivo*. **(A)** Representative Western blot and analysis of NLRP3 in doxorubicin (DOX)-treated hearts (n = 6). **(B)** Representative Western blot and analysis of TLR4 in DOX-treated hearts (n = 6). **(C)** Representative Western blot and analysis of NLRP3 in DOX-treated H9c2 cells (n = 6). **(D)** Representative Western blot and analysis of TLR4 in DOX-treated H9c2 cells (n = 6). For panels **(A**, **B)**, *P < 0.05 versus NS + Ad-GFP, #*P* < 0.05 versus DOX + Ad-GFP. For panels **(C**, **D)**, *P < 0.05 versus PBS + Ad-GFP, #*P* < 0.05 versus DOX + Ad-GFP.

It was reported that TLR4 and downstream signal NLRP3 were crucial in cardiac injury; thus, the activation levels of TLR4 and NLRP3 were detected. Our data showed that NLRP3 and TLR4 protein expression were both increased by DOX and were significantly decreased in Hsp22-overexpressing mice (**Figures 4A, B**). We solidify our evidence *in vitro* by using the H9c2 cell line. As a result, the increased NLRP3 and TLR4 levels induced by DOX treatment were reduced by overexpression of Hsp22 (**Figures 4C, D**).

### Hsp22 Exerted a Protective Effect by Regulating NLRP3 Signaling

**Figure 5:**
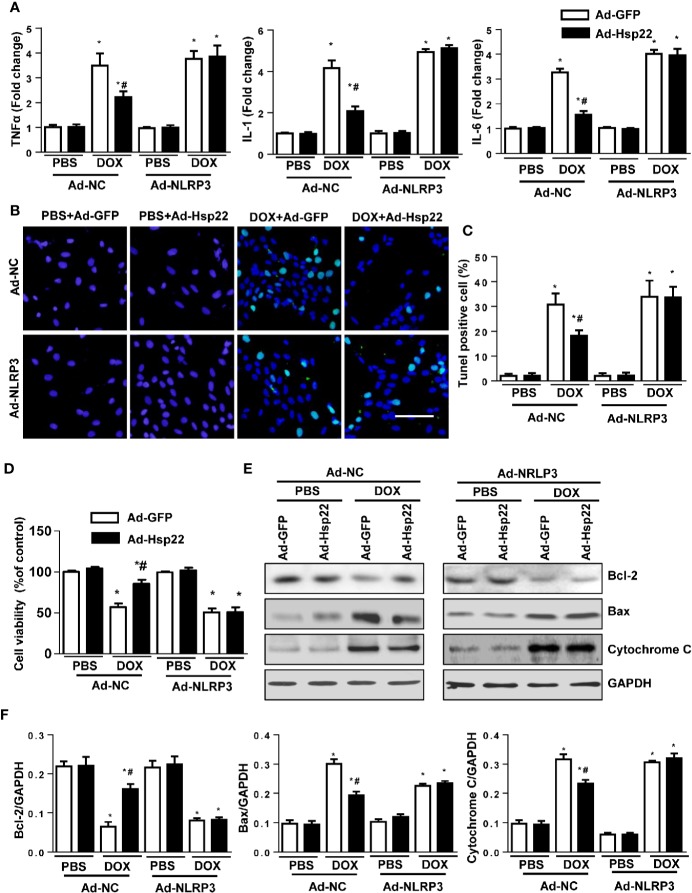
Heat shock protein 22 (Hsp22) inhibited doxorubicin (DOX)-induced inflammation and cell loss *via* inhibition of the activation of the NOD-, LRR-, and Pyrin Domain-Containing Protein 3 (NLRP3). **(A)** The mRNA levels of inflammation-related genes in the indicated groups (n = 4). **(B, C)** Terminal deoxynucleotidyl transferase dUTP nick end labeling (TUNEL) staining and the quantitative results in the indicated groups (n = 4). **(D)** Cell viability after the treatment with DOX in the indicated groups (n = 4). **(E, F)** Representative Western blot and analysis of Bcl-2, Bax, and cytochrome C in the indicated groups (n = 4). *P < 0.05 versus PBS + Ad-NC group, #*P* < 0.05 versus the DOX + Ad-GFP group.

To assess the role of NLRP3 on Hsp22-mediated protection, we coinfected H9c2 cells with Ad-Hsp22 plus Ad-NLRP3, followed by the addition of DOX for 12 h. As shown in **Figure 5A**, the increased pro-inflammatory cytokines by DOX treatment were reduced by Hsp22 overexpression. Interestingly, we observed that suppression of pro-inflammatory cytokines by overexpression of Hsp22 was blocked by increased NLRP3 expression. Then, we evaluated the severity of DOX-induced cardiomyocyte apoptosis. Hsp22 decreased DOX-induced cell loss *in vitro*, as indicated by TUNEL staining and cell viability. But overexpression of NLRP3 attenuated the protective effect of Hsp22 on apoptosis (**Figures 5B–D**). DOX treatment decreased Bcl-2, but increased Bax and cytochrome C in cardiomyocytes protein level, while these were reversed in Hsp22-overexpressing cells. Whereas overexpression of NLRP3 undermined the protective effect of Hsp22 (**Figures 5E, F**). The above findings demonstrate that the beneficial role of Hsp22 in DOX-induced cardiomyocyte apoptosis is dependent on the regulation of NLRP3.

## Discussion

Although no consensus has been reached, the progressive elucidation of the molecular mechanisms by which Hsp22 promotes cardiac protection is still encouraging. Our study is the first to find that DOX can increase the protein expression level of Hsp22 in C57BL/6 mice, and the overexpression of Hsp22 can reduce the cardiac dysfunction induced by DOX, the mechanism of which is related to downregulating the inflammatory response and reducing the apoptosis of cardiomyocytes. Further studies revealed that these protective effects were mediated by inhibition of TLR4/NLRP3 in C57BL/6 mice and in H9c2 cells, while overexpression of NLRP3 can eliminate Hsp22-mediated cardiac protection. Our research provides a new method for the treatment of cardiac injury caused by DOX.

As one of the important molecular chaperones, the protective effects of Hsps on ischemia/reperfusion injury and other stress stimuli have been studied comprehensively and systematically (Fan et al., 2005; Li et al., 2013); however, there has not been much concern about their protective effect on DOX-induced cardiotoxicity and underlying mechanisms. We noted a recent published study showing that increased levels of Hsp27 in cardiomyocytes resulted in more resistance to DOX-induced cell death, and therefore, DOX-induced cardiac dysfunction and animal mortality were significantly reduced (Bernard et al., 2011). Other studies further demonstrated that Hsp20 could reduce DOX-triggered oxidative stress and cardiotoxicity through regulating the AKT pathway (Fan et al., 2008). Consistent with the previous studies, our present study found that Hsp22 protein level was decreased after DOX treatment in C57BL/6 mice; there is some potential for Hsp22 to be a defender in the treatment of DOX-induced cardiomyopathy.

One of the main mechanisms of DOX-induced cardiac injury is excessive inflammation. DOX treatment can activate the cardiac NF-κB signaling pathway, which promotes the activation of inflammatory cells and the release of pro-inflammatory factors, leading to cascade amplification of inflammation and ultimately induce cardiac dysfunction (Liu et al., 2009). A study has found that in human leptomeningeal smooth muscle cells and human brain astrocytes, Hsp22 could increase the level of IL-8, soluble intercellular adhesion molecule 1 (ICAM-1), and monocyte chemoattractant protein 1 (Bruinsma et al., 2011). Therefore, in this study, we also explored the effect of Hsp22 on DOX-induced cardiac inflammation. Our results show that Hsp22 can reduce the activation of NF-κB in cardiomyocytes and its nuclear translocation in cardiomyocytes of C57BL/6 mice, thereby diminishing the synthesis and release of pro-inflammatory factors, which might ultimately reduce the inflammatory response of the cardiomyocytes.

In addition, TLRs are involved in the activation of innate immunity and also the important components in the cardiac stress response. TLR4 is the first and very important mammalian Toll protein to be identified (Yousif and Al-Amran, 2011). A growing number of studies suggest that TLR4 plays a role in heart damage caused by DOX. Riad et al. (2008) found that deletion of TLR4 significantly improves DOX-induced cardiac dysfunction. TLR4 deficiency could protect against DOX-induced cell apoptosis, which exerts a cardioprotective function (Yao et al., 2012). In line with previous studies, we found that TLR4 was elevated after DOX treatment in both mice heart and cardiomyocytes. NLRP3, a multi-protein complex, has been reported to be a target of TLR4. Studies showed that NLRP3 deficiency reduces the inflammatory response and protects against DOX-induced cardiac injury (Kobayashi et al., 2016). Our study found that NLRP3 was activated during DOX treatment, and overexpression of NLRP3 completely abolished the inhibition effect of Hsp22 on the pro-inflammatory cytokines. Our work implies that Hsp22 could suppress DOX-induced cardiac injury *via* regulating TLR4/NLRP3.

Cell apoptosis is the main feature of DOX-induced cardiac injury. It has been found that Hsp22 possesses anti-apoptosis activities in melanoma, glioblastoma, or breast cancer cells (Shemetov et al., 2008). Furthermore, it has been suggested in ischemic myocardium that Hsp22 reduces cardiac ischemic injury by pre-activation of the heart’s anti-apoptotic pathway and adaptive metabolic changes (Depre et al., 2006). Consistent with these reports, we demonstrated that Hsp22 inhibited DOX-induced cell loss in C57BL/6 mice and in H9c2 cells. Our present study showed that overexpression of NLRP3 almost completely abolished Hsp22-mediated anti-apoptosis effect, further implying the key role of TLR4/NLRP3 in cardioprotection by Hsp22.

This study demonstrates that Hsp22 improves acute DOX-induced cardiac injury by reducing cardiac inflammation and cardiomyocyte apoptosis in mice and H9c2 cells. Certainly, more experimental data are needed to accurately explain the cardioprotective effect of Hsp22. Altogether, our observations provide a novel potential therapeutic target for cardiac injury caused by DOX.

## Data Availability Statement

The raw data supporting the conclusions of this article will be made available by the authors, without undue reservation, to any qualified researcher.

## Ethics Statement

The animal study was reviewed and approved by The Ethics Committee of Union Hospital, Huazhong University of Science and Technology.

## Author Contributions

YL performed the experiments, YW analyzed the data, KH wrote the paper, and QZ designed the experiments.

## Funding

This study was supported by the National Nature Science Foundation of China (81400323 to KH).

## Conflict of Interest

The authors declare that the research was conducted in the absence of any commercial or financial relationships that could be construed as a potential conflict of interest.
